# Supplementary material for: Pervasive Transcription-coupled DNA repair in E. coli
Source: Nat Commun. 2022 Mar 30;13:1702. doi: 10.1038/s41467-022-28871-y (PMC8967931; doi:10.1038/s41467-022-28871-y)
Supplement: Supplementary file 1 — Supplementary Information [file 41467_2022_28871_MOESM1_ESM.pdf]

SUPPLEMENTARY INFORMATION

Pervasive Transcription-coupled DNA repair in *E. coli*

Britney Martinez[1], Binod K. Bharati[1,2], Vitaly Epshtein[1], and Evgeny Nudler[1,2]

[1]Department of Biochemistry and Molecular Pharmacology, New York University School of Medicine, New York, NY, 10016, USA

[2]Howard Hughes Medical Institute, New York University School of Medicine, New York, New York 10016, USA

Content:

Supplementary Tables 1-4

SI Table 1: List of strains used in this study.
SI Table 2: Oligonucleotides used for CPD-seq library construction.
SI Table 3: qPCR primers used in this study.
SI Table 4: DNA templates used for the *in vitro* transcription assays.

Supplementary Figures 1-9

SI Fig.1: Distribution of CPD-lesion accumulation after UV damage.
SI Fig.2: Quantitation of NER proteins before and after Rifampicin treatment.
SI Fig.3: Metanalysis windows in genes with an antisense transcription preference at their gene end.
SI Fig.4: Global antitermination increases sense transcription.
SI Fig.5: *rho15* mutant increases repair in NTS regions with increased readthrough after Rho inhibition.
SI Fig.6: Confirmation of Rho-dependence at chromosomal terminators.
SI Fig.7: UV increases transcriptional readthrough at Rho termination sites.
SI Fig.8: Mfd promotes repair only in highly transcribed regions.
SI Fig.9: Pervasive TCR model.

Supplementary Table 1. List of strains used in this study

**Strain**	**Genotype**	**Source**
MG1655	wildtype	Lab stock
MG1655- Δ*uvrA*	MG1655- Δ*uvrA::Cm*^{r}	Lab stock
MG1655- Δ*uvrD*	MG1655- Δ*uvrD::Km*^{r}	Lab stock
MG1655 Δ*mfd*	MG1655 *Δmfd::Cm*^{r}	Lab stock
BM1001	MG1655 *rho*-L187R	This study
BM1002	MG1655 *rho*-D210G	This study
MG1655-*rho15*	MG1655 *rho15*(Ts)	Lab stock
MG1655 *lexA3*	MG1655 *lexA3:: Km*^{r}	Lab stock
BK1001	MG1655-UvrA-FLAG*::Km*^{r}	Bharati et al.
BK1002	MG1655-UvrB-FLAG*::Km*^{r}	Bharati et al.
BK1003	MG1655-UvrC-FLAG*::Km*^{r}	Bharati et al.
BK1004	MG1655-UvrD-FLAG*::Km*^{r}	Lab stock
UvrAB-OverExpression		
p-Mfd	pCA24N-mfd	ASKA (M. Kitagawa *et al)*
pCA24N	pCA24N	ASKA (M. Kitagawa *et al)*

Supplementary Table 2. Oligonucleotides used for CPD-seq library construction

Name	Sequence
A1_T	5'-ACACTCTTTCCCTACACGACGCTCTTCCGATC-phosphorothioate-T-3'
A1_B	5'-phosphate-GATCGGAAGAGCGTCGTGTAGGGAAAGAGTGTC-dideoxy-3'
A2_T	5'-phosphate-AGATCGGAAGAGCACACGTCTGAACTCCAGTCAC-dideoxy-3'
A2_B	5'-biotin-GGTGACTGGAGTTCAGACGTGTGCTCTTCCGATCTNNNNNN-C3

Supplementary Table 3. qPCR primers used in this study

Primer Sequence	Gene	Postition	Direction
gtggattcgtccgctgttta	ProA	Before rho terminator	Forward
ttgtaagtggtcagtgcttcc	ProA	Before rho terminator	Reverse
gccgatatagctcagttggtag	ProA	After rho terminator	Forward
aatggtgccgataataggagtc	ProA	After rho terminator	Reverse
gcgctttgtgaaagtgaatacc	TrxC	Before rho terminator	Forward
ttcggtactgcgccattaag	TrxC	Before rho terminator	Reverse
aacgctgttctccagttacg	TrxC	After rho terminator	Forward
cagcgacgaacgcgattac	TrxC	After rho terminator	Reverse
atggttcgtttcccggaatg	DsbB	Before rho terminator	Forward
gcggcatttccagacctaaa	DsbB	Before rho terminator	Reverse
gtataacgtggtgaaagcatgg	DsbB	After rho terminator	Forward
gggcagaggaacactctattt	DsbB	After rho terminator	Reverse

Supplementary Table 4. **DNA templates used for the *in vitro* transcription assays**

Name	Sequence
A1-proA T	*tccagatcccgaaaatttatcaaaaagagtattgacttaaagtctaacctataggatacttacagcc*<br>ATCGAGAGGGACACGGCGAAataaaaccgggtgatgcaaaagtag**ccatttgattcacaaggccattgacgcatcgcccggttagttttaaccttgtccaccgtgattcacgttcgtg**aacatgtcctttcagggccgatatagctcagttggtagagcagcgcattcgtaatgcgaaggtcgtaggttcgactcctattatcggcaccattaaaatcaaattgttacgtaagatcttatcattctcccaccaaaaaattatcttaatgtaacagctggtgtaagtaaattctatcaacgaagatcaatcttatctactgaccaaaaaggcctgatagggcttcgctcactatacatccttggctgcaggtttagttgtacaccactcctaaatttaatgtgttggcaatgtgttcaataaagctcgaacaaattagctcattatgatcggttaatacttcaacttctggttgcatgattgtttgtccgtaaaaagataacgcgcctgccgggtagtagcaggcgcattacgcaataggtaaacaagggaggaagttcagaaatgtaaatcgggaaggttgtacgcaatgttcatcgtactacgttgttacggctttgcc
A1 dsbD T	*tccagatcccgaaaatttatcaaaaagagtattgacttaaagtctaacctataggatacttacagcc*<br>ATCGAGAGGGACACGGCGAA**gtcgctaatccattcggcgctcctgcgggagcgctttttcctgccgctatatttatttgaccctcagtaaa**tcagaacttcgcgttgtataacgtggtgaaagcatggctcgcttcatctgccattgttgctggatcccctgcccggcaaaatagagtgttcctctgccctctttagcattcagtgtatccattaccgtcatcaattgctcactcccggggcgcggtgcgttgtcatcgaataaattgagctgcgcgactccctgactgaagaaatcccccagcatcacgcccgctttttggtaacgatggcccgcttgccagatggcatccagagatcgcgtagcagcgttaatgatatccctgctgtcctgagtgggcgtcagcagttttaccgacgcgctattgccgtaataaggttcattgagcgcaaatggtgacgtcttaataaacgtggagataaaccgacaatattgatgctcgctgcgaagtttttccgccgcccgggcagcgtaactacaaatggcctgccgcatcgacggataat
A1-trxA T	*tccagatcccgaaaatttatcaaaaagagtattgacttaaagtctaacctataggatacttacagcc*<br>ATCGAGAGGGACACGGCGAActtaatggcgcagtaccgaaagcgccgttcgatagctggctgaacgaatc**tctttaatcttaccggggcgcatcttgtgccccgttttctcctctgcgacaatggcgtttttttcgacgctc**tcttatgaccgaaaacgctgttctccagttacgcgccgagcgtattgcgcgcgcaacacgtccttttcttgcccgcggtaatcgcgttcgtcgctgccaacgctgtcttttgccagagaaattatgtctctgttcg

Non-transcribed part of T7A1 promoter sequence is italicized, the transcribed part of T7A1 sequence is capitalized, Rho utilization sequences are shown in bold.

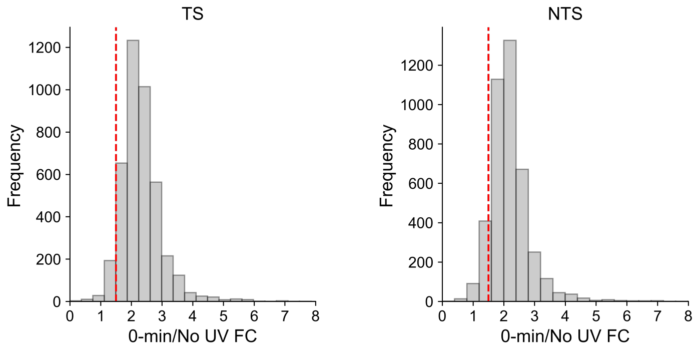

Supplementary Fig. 1. Distribution of CPD-lesion accumulation after UV damage. A histogram showing the distribution of the fold change in normalized TT-CPDs in the 0-min timepoint over the No UV timepoint in WT cells. The red dashed line marks a 1.5-fold increase in CPD-lesions.

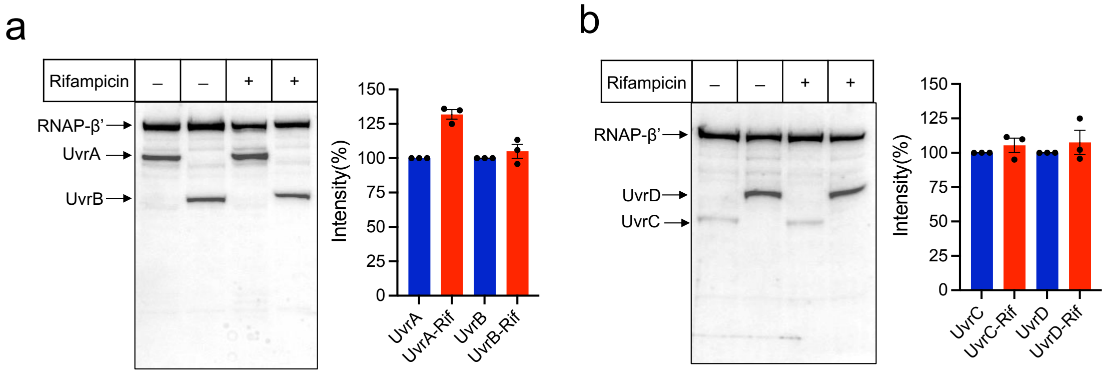

Supplementary Fig. 2. Quantitation of NER proteins before and after rifampicin treatment. Representative Western blots of the intracellular **a**, UvrA, UvrB and **b**, UvrD, UvrC proteins. The bar chart on the right represents the quantitation of intracellular UvrABCD proteins. Data are the mean ± SEM from at least three independent experiments (n=3).

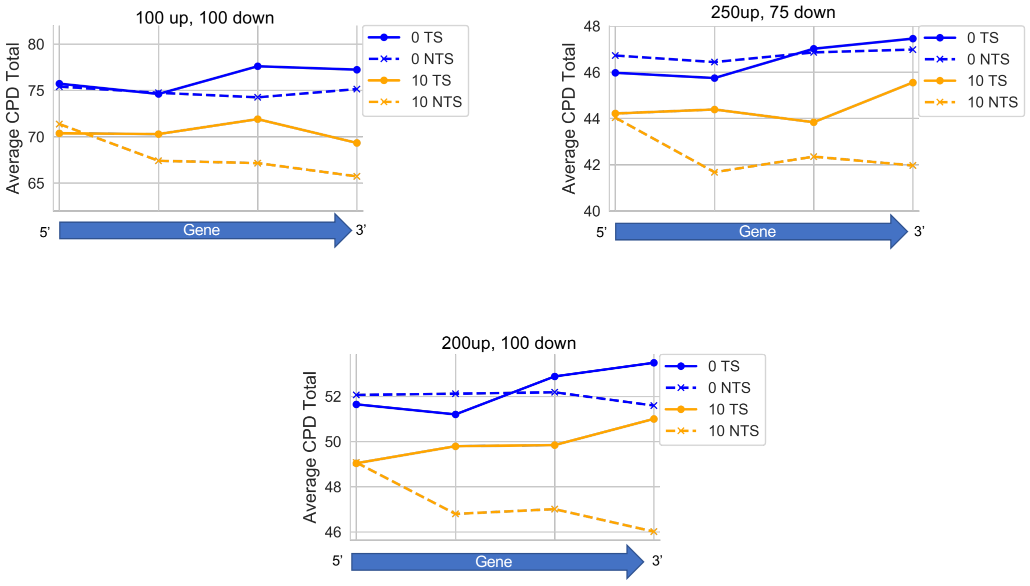

Supplementary Fig. 3. Metanalysis windows in genes with an antisense transcription preference at their gene end. Three different gene end windows were used to obtain genes that had an antisense transcription preference within these windows. The up and downstream coordinates were determined based on the end of the open reading frame. Metanalysis plots shows the TT-CPD total in the NT and 10-min timepoint and each point represents of a quarter of the gene. The following number of genes were contained in each analysis: top left: 364, top right: 252, and bottom center: 272.

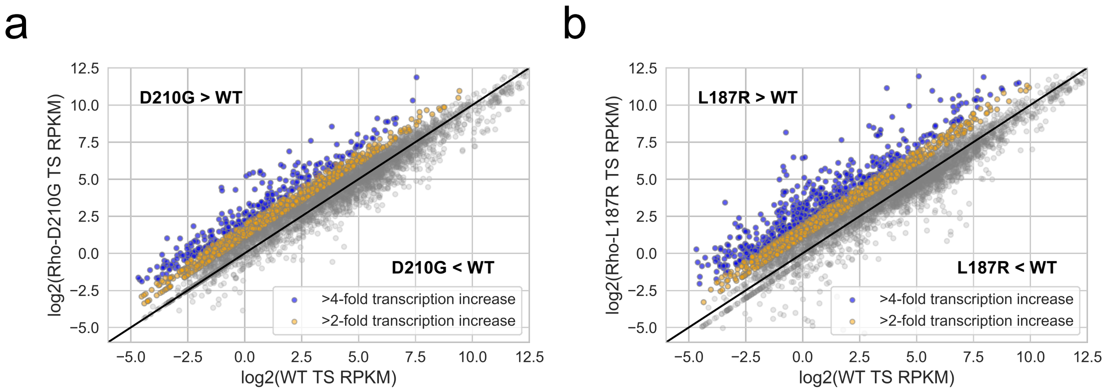

Supplementary Fig. 4. Global antitermination increases sense transcription. Scatterplots comparing the TS RPKM (RNA-seq) in WT vs Rho^{L187R} (top) or Rho^{D210G} (bottom) mutants. Genes are colored based on their fold increase in the Rho mutant compared to WT. Blue genes had a greater than 4-fold increase in antisense transcription and yellow genes had a 2-4-fold increase in sense transcription.

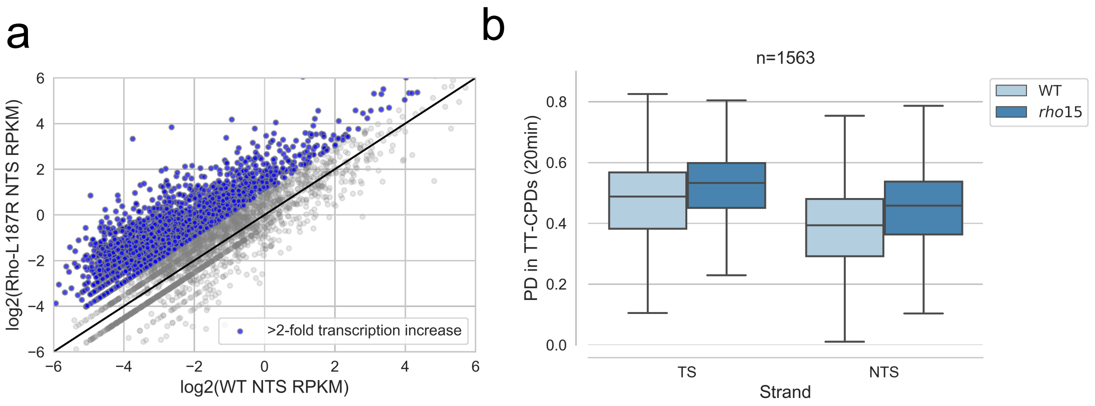

Supplementary Fig. 5. *rho15* mutant increases repair in NTS regions with increased readthrough after Rho inhibition. a, Rho-L187R NTS RPKM vs WT NTS RPKM. Genes with at least 2-fold increase in mutant are marked in blue and were used in *rho15* repair analysis. **b,** The recovery of TT-CPD lesions in WT and *rho15* mutant strains in genes that had increased readthrough in the Rho-L187R mutant. The box shows the interquartile range (IQR), the line shows the median, and the whiskers extend to 1.5xIQR. The top of the box represents the 75th percentile and bottom represents the 25th percentile. n denotes the number of genes in each plot.

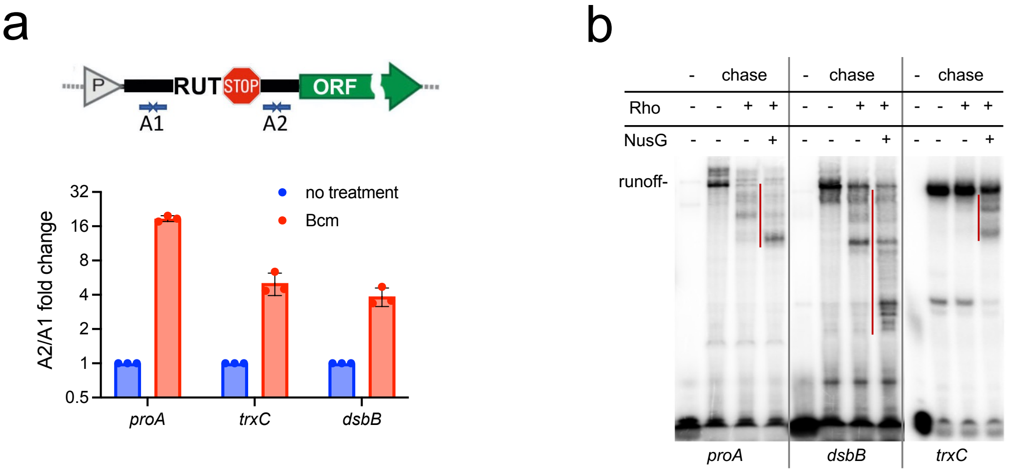

Supplementary Fig. 6. Validation of Rho-dependent terminators. a, Schematic showing the location of the qPCR primers A1 and A2 relative to the RUT and termination site (top). The A1/A2 fold-change at three different native Rho terminators after BCM (50 µg/mL) treatment (bottom). The data are means ± SD of 3 (*proA*), 3 (*trxC*), and 4(*dsbB*) independent replicates. **b,** *In vitro* transcription showing Rho-dependent termination of three chosen terminators (red lines mark Rho termination bands). The gel is a representation of at least 3 independent experiments with similar results.

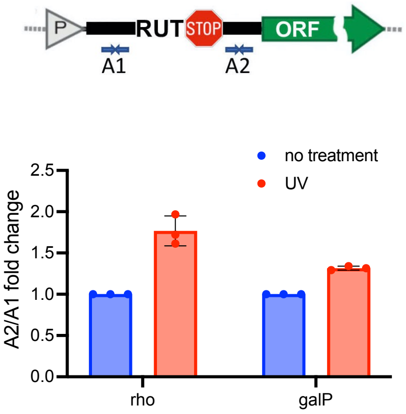

Supplementary Fig. 7. UV increases transcriptional readthrough at Rho termination sites. Schematic showing the location of the qPCR primers A1 and A2 relative to the RUT and termination site (top). The A1/A2 fold-change at two different native Rho terminators after UV exposure (bottom). The data are means ± SEM of 3 (*rho*) and 2 (*galP*) independent replicates.

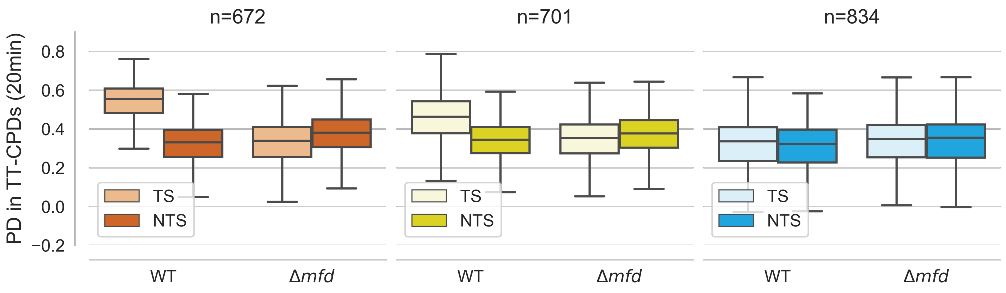

Supplementary Fig. 8. Mfd promotes repair only in highly transcribed regions. The recovery of TT-CPD lesions at the 20-min recovery timepoint in WT and Δ*mfd* cells. Genes were split by transcription level based on RNA-seq data (High=orange, Mid=yellow, and Low=blue). The box shows the interquartile range (IQR), the line shows the median, and the whiskers extend to 1.5xIQR. The top of the box represents the 75th percentile and bottom represents the 25th percentile. n denotes the number of genes in each plot.

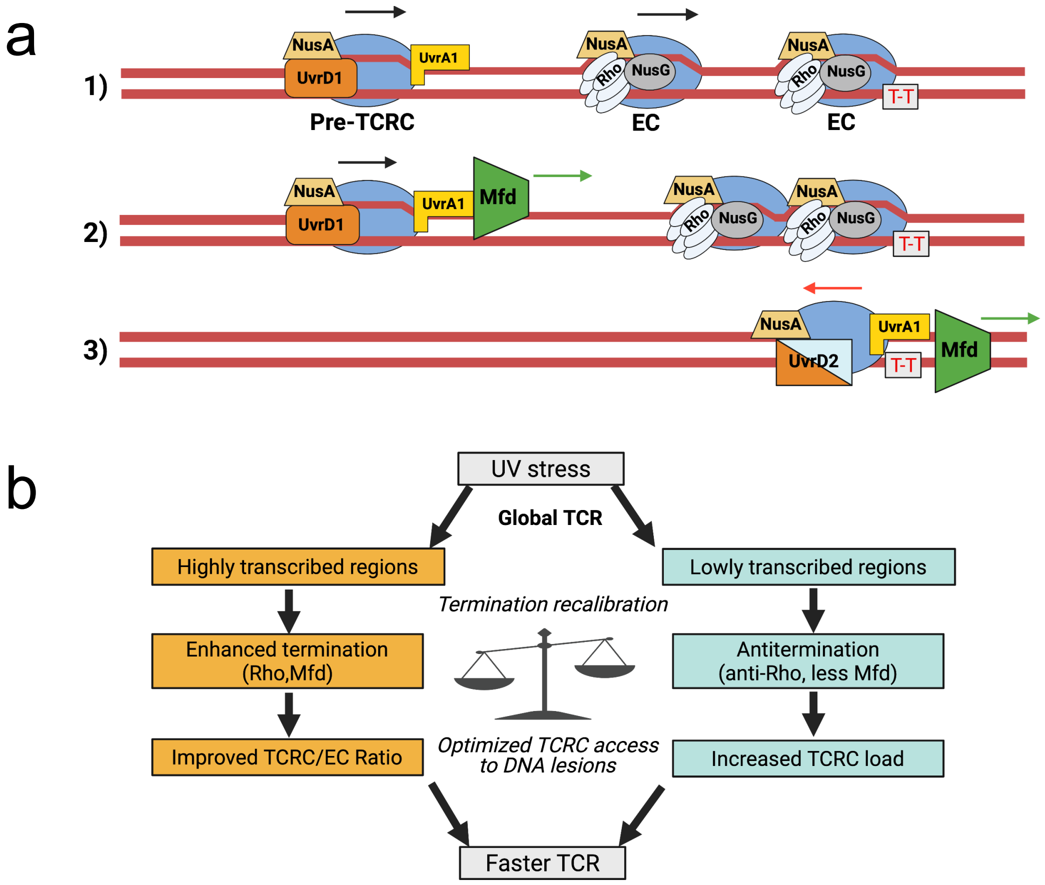

Supplementary Fig. 9. Pervasive TCR model. a, Termination factors Mfd and Rho help to clear the path to the lesion sites by TCRC. 1) Highly transcribed genes are more likely to accumulate arrays of elongation complexes (ECs) that create a roadblock against RNAPs equipped with NER machinery, i.e. pre-TCRCs and TCRCs (pre-TCRC is shown). 2) UvrA on the pre-TCRC helps to recruit Mfd (**Bharati et al**)[41]. 3) Mfd then processively terminates the array of ECs so that the pre-TCRCs/TCRCs can access the lesion. **b**, The concept of pervasive TCR. Based on the evidence presented we propose that every part of the genome must be transcribed in order to enable NER. Towards this goal, transcription termination has to be recalibrated to optimize TCRC access to the lesions genome-wide. Due to highly efficient transcription termination, RNAP is sparse in NTS and intergenic regions. The TS of low transcribed genes would also have low RNAP presence due to lower transcription initiation. The low frequency of RNAP in these regions results in slow lesion detection and less efficient NER than regions with high RNAP presence. However, TCR is still able to occur in these regions due to pervasive transcription. Pre-TCRCs are likely to be more resistant to Rho-dependent termination (see Discussion) and are therefore more likely to transcribe past termination sites and into low transcribed regions. Increasing global antitermination increases the number of ECs and Pre-TCRCs in areas that were previously low transcribed. ECs have the potential to be converted to Pre-TCRCs. Therefore, the overall increase in RNAP presence results in faster repair. In the highly transcribed genes, the situation is opposite (shown in a). Therefore, the enhanced termination by Mfd and Rho benefits TCR.
