# Supplementary material for: Pervasive Transcription-coupled DNA repair in E. coli
Source: Nat Commun. 2022 Mar 30;13:1702. doi: 10.1038/s41467-022-28871-y (PMC8967931; doi:10.1038/s41467-022-28871-y)
Supplement: Supplementary file 3 — Reporting Summary [file 41467_2022_28871_MOESM3_ESM.pdf]

Corresponding author(s): Evgeny Nudler

Last updated by author(s): Feb 8, 2022

Reporting Summary

Nature Portfolio wishes to improve the reproducibility of the work that we publish. This form provides structure for consistency and transparency in reporting. For further information on Nature Portfolio policies, see our Editorial Policies and the Editorial Policy Checklist.

Statistics

For all statistical analyses, confirm that the following items are present in the figure legend, table legend, main text, or Methods section.

n/a	Confirmed	
☐	☒	The exact sample size (*n*) for each experimental group/condition, given as a discrete number and unit of measurement
☐	☒	A statement on whether measurements were taken from distinct samples or whether the same sample was measured repeatedly
☐	☒	The statistical test(s) used AND whether they are one- or two-sided<br>*Only common tests should be described solely by name; describe more complex techniques in the Methods section.*
☐	☒	A description of all covariates tested
☒	☐	A description of any assumptions or corrections, such as tests of normality and adjustment for multiple comparisons
☐	☒	A full description of the statistical parameters including central tendency (e.g. means) or other basic estimates (e.g. regression coefficient) AND variation (e.g. standard deviation) or associated estimates of uncertainty (e.g. confidence intervals)
☐	☒	For null hypothesis testing, the test statistic (e.g. *F*, *t*, *r*) with confidence intervals, effect sizes, degrees of freedom and *P* value noted<br>*Give P values as exact values whenever suitable.*
☒	☐	For Bayesian analysis, information on the choice of priors and Markov chain Monte Carlo settings
☒	☐	For hierarchical and complex designs, identification of the appropriate level for tests and full reporting of outcomes
☒	☐	Estimates of effect sizes (e.g. Cohen's *d*, Pearson's *r*), indicating how they were calculated

Our web collection on statistics for biologists contains articles on many of the points above.

Software and code

Policy information about availability of computer code

Data collection	No software was used
Data analysis	Sequencing reads were aligned using Bowtie2 (v 2.4.5). The aligned reads were then split based on whether they were in the forward or reverse orientation with Samtools (v 1.14). HTseq (v 1.99.2) was used to determine the amount of reads that mapped to each gene and Python scripts (https://zenodo.org/record/5979538) were used to generate the final count tables of reads per strand for each gene. The Reads Per Kilobase Million (RPKM) was then calculated for each sample using these counts. Readthrough analysis was done using bedtools (v 2.30.0) genomecov to determine the read coverage across the entire E. coli genome. These counts were then normalized by the total number of aligned reads per million (RPM).

For manuscripts utilizing custom algorithms or software that are central to the research but not yet described in published literature, software must be made available to editors and reviewers. We strongly encourage code deposition in a community repository (e.g. GitHub). See the Nature Portfolio guidelines for submitting code & software for further information.

Data

Policy information about availability of data

All manuscripts must include a data availability statement. This statement should provide the following information, where applicable:
- Accession codes, unique identifiers, or web links for publicly available datasets
- A description of any restrictions on data availability
- For clinical datasets or third party data, please ensure that the statement adheres to our policy

The DNA and RNA sequencing data generated in this study have been deposited to the Gene Expression Omnibus (GEO) under the accession code GSE17192 [https://www.ncbi.nlm.nih.gov/geo/query/acc.cgi?acc=GSE171972]. The qPCR data generated in this study are provided in the Source Data File.

Field-specific reporting

Please select the one below that is the best fit for your research. If you are not sure, read the appropriate sections before making your selection.

☒ Life sciences ☐ Behavioural & social sciences ☐ Ecological, evolutionary & environmental sciences

For a reference copy of the document with all sections, see nature.com/documents/nr-reporting-summary-flat.pdf

Life sciences study design

All studies must disclose on these points even when the disclosure is negative.

Sample size	Each experiment had one sample. No statistical test was used to determine the sample size and sample sizes were determined by sequencing capacities.
Data exclusions	No data was excluded.
Replication	The WT CPD-seq data was replicated for each new sequencing run and other mutants had CPD-seq data performed once. The RNA-seq data is from a single experiment. The qPCR experiments were performed in at least duplicates. The Western blot analysis was performed in triplicates.
Randomization	Samples run on different sequencing runs were split based on the order in which the experiments were performed. If one experiment exceeded the sequencing capacity then groups were randomly assigned to a certain run.
Blinding	Blinding was not performed because one person collected and analyzed the data.

Reporting for specific materials, systems and methods

We require information from authors about some types of materials, experimental systems and methods used in many studies. Here, indicate whether each material, system or method listed is relevant to your study. If you are not sure if a list item applies to your research, read the appropriate section before selecting a response.

Materials & experimental systems

n/a	Involved in the study	
☐	☒	Antibodies
☒	☐	Eukaryotic cell lines
☒	☐	Palaeontology and archaeology
☒	☐	Animals and other organisms
☒	☐	Human research participants
☒	☐	Clinical data
☒	☐	Dual use research of concern

Methods

n/a	Involved in the study	
☒	☐	ChIP-seq
☒	☐	Flow cytometry
☒	☐	MRI-based neuroimaging

Antibodies

Antibodies used	Monoclonal anti-FLAG M2 (Sigma-Aldrich; F1804, Lot# SLCF 4933), Anti-6X His tag (Abcam; Ab9108, Lot# GR3345213-2), Donkey anti-mouse(AlexaFluor 488; Invitrogen, A-21202, lot# 2018296), and Donkey anti-rabbit (Alexa Fluor 647; Invitrogen, A-31573, Lot# 2083195) antibodies were used following the information provided at manufacturers websites of the respective antibodies.
Validation	Antibodies were validated using Western-Blot analysis following the manufacturer's protocol with details provided in the Method section.
